# What works in ‘real life’ to facilitate home deaths and fewer hospital admissions for those at end of life?: results from a realist evaluation of new palliative care services in two English counties

**DOI:** 10.1186/1472-684X-13-37

**Published:** 2014-07-28

**Authors:** Lesley Wye, Gemma Lasseter, John Percival, Lorna Duncan, Bethany Simmonds, Sarah Purdy

**Affiliations:** 1 Centre for Academic Primary Care, University of Bristol, Canynge Hall, 39 Whatley Road, Bristol BS8 2PS, UK

## Abstract

**Background:**

We evaluated end of life care services in two English counties including: coordination centres, telephone advice line, ‘Discharge in Reach’ nurses, a specialist community personal care team and community nurse educators. Elsewhere, we published findings detailing high family carer satisfaction and fewer hospital admissions, Accident and Emergency attendances and hospital deaths for service users compared to controls. The aim of this paper is to discuss what contributed to those outcomes.

**Methods:**

Using realist evaluation, data collection included documentation (e.g. referral databases), 15 observations of services and interviews with 43 family carers and 105 professionals. Data were analysed using framework analysis, applying realist evaluation concepts. Findings were discussed at successive team meetings and further data was collected until team consensus was reached.

**Results:**

Services ‘worked’ primarily for those with cancer with ‘fast track’ funding who were close to death. Factors contributing to success included services staffed with experienced palliative care professionals with dedicated (and sufficient) time for difficult conversations with family carers, patients and/or clinical colleagues about death and the practicalities of caring for the dying. Using their formal and informal knowledge of the local healthcare system, they accessed community resources to support homecare and delivered excellent services. This engendered confidence and reassurance for staff, family carers and patients, possibly contributing to less hospital admissions and A&E attendances and more home deaths.

**Conclusions:**

With demand for 24-hour end of life care growing and care provision fragmented across health and social care boundaries, services like these that cut across organisational sectors may become more important. They offer an overview to help navigate those desiring a home death through the system.

## Background

A major focus of the English national End of Life Care strategy is helping people to reduce their use of hospital services and die in community settings [1]. The launch of this strategy has resulted in a focus on improving the quality and quantity of end of life care provision across the United Kingdom [2]. Improving end of life care is a key component of national policy making and one of the twelve key work streams of the QIPP agenda (Quality, Innovation, Productivity and Prevention) that aims to reduce the National Health Service (NHS) budget by £20 billion by 2015 [3]. But to identify what makes a difference, we need more ‘real life’ service evaluations of end of life care provision, given that research “is too slow, too expensive and frequently does not come up with results which are useful for policy makers and commissioners” [1].

To address some of the difficulties in end of life care research, the MOREcare project was set up by academics and others interested in end of life care

“to identify, appraise and synthesise ‘best practice’ methods to develop and evaluate palliative and End of Life Care, particularly focusing on complex service-delivery interventions and reconfigurations” [4]

In 2013, the MOREcare project called for evaluations of palliative services to include information on interventions, the local context and intended/unintended outcomes [5,6]. Realist evaluation, with its emphasis on context, ‘mechanism’ and outcomes [7] may be one possible way forward in addressing the evaluation ideals set out by MOREcare. But few applied examples of realist evaluation exist, as a recent review found only 20 published studies to date [8]. The purpose of this paper is to add to the evidence base of applied examples by presenting findings from a realist evaluation of a major service re-configuration of end of life care services known as ‘Delivering Choice’.

### Delivering choice programme

Marie Curie Cancer Care’s Delivering Choice Programme (DCP) has 19 projects running across England and Scotland. A key objective of Delivering Choice is working in partnership with the local providers and commissioners to develop 24-hour services to meet local needs [9]. One project ran from 2008 – 2011 in two southern counties in England.

North Somerset had about 2000 palliative deaths a year from a mixed urban/rural population. Quantitative data from the six month study period (1 September 2011 to 29 February 2012) showed that of those who died in North Somerset, 46% were men and 54% were women, 84% were aged over 70 and the most common causes of death were cancer (28%), heart disease (18%), respiratory disease (15%) and dementia (15%). Thirty eight percent of the study population died in hospital. Somerset had about 5000 palliative deaths a year amongst a largely rural population. Of those who died during the six month study period in Somerset, 45% were men and 55% were women, 85% were aged over 70 and the most common causes of death were cancer (29%), heart disease (18%), respiratory disease (13%) and dementia (13%). Thirty six percent of the study population died in hospital [10].

Delivering Choice services were developed by local professionals from the NHS and local authorities, clinicians and managers from the acute, primary and community sectors, hospice staff and a small local Marie Curie Cancer Care funded team. Services launched in 2010 or 2011 included:

• Two End of Life Care facilitators who worked across the county. These facilitators had specialist palliative community nursing experience and educated care home staff and community and hospice nurses to improve end of life care skills and knowledge. They did not work directly with patients. (North Somerset only)

• Two End of Life Care Coordination Centres (one in each county) that organised packages of care. These packages of care included equipment, night staff and personal care staff. In Somerset, the coordination centre bought all support needed from external care providers (e.g. personal care staff) and was run by a nurse manager with administrators. In North Somerset, the coordination centre was located on social service premises and offered a ‘one stop shop’ where all needs could be met (i.e. financial assessment, personal care and equipment provision). This Coordination Centre included an in-house personal care team and was run by a nurse manager supported by two specialist end of life care nurses and administrators.

• An Out of Hours Advice and Response Line delivered by a local hospice and manned by specialist palliative care nurses. They responded to calls from professionals, family carers and patients. (Somerset only)

• Two End of Life Care ‘Discharge in Reach’ nurses based in two different hospitals in Medical Admissions Units and Emergency Departments. They identified patients wanting to die in the community and facilitated fast discharges. (Somerset only)

These services were supported by a named ‘Key Worker’ and an electronic end of life care register (ADASTRA). The aim of the (ADASTRA) electronic end of life care register was to allow professionals across different organisations (e.g. community nurses, hospital staff, voluntary sector professionals, paramedics) to access details on preferred place of death and other data. Table 1 details the Delivering Choice interventions. Findings related to the specialised personal support team [11] are published elsewhere (Table 1).

**Table 1:** Delivering choice interventions

**North Somerset**	**Somerset**
Electronic end of life care register	Electronic end of life care register
Key Worker	Key Worker
End of Life Care facilitators	Discharge in Reach nursing service
Coordination centre with integrated personal care team	Coordination centre
	Out of hours response and advice telephone line

All palliative patients were eligible to access the services across the two counties; the services were not only for cancer patients. The underlying theory behind Delivering Choice was that the provision of advice, information and support, whether emotional or practical (e.g. equipment, night sitters) would lead to a reduction in hospital utilisation and an increase in deaths in the community. As this ‘real life’ evaluation took place in a naturalistic setting, patients were not randomly allocated to any particular service. Instead, patients were identified by the service providers (Discharge in Reach nurses), were referred by a healthcare professional (Coordination Centres) or initiated contact themselves after having received information about the service (Out of Hours advice line).

### Evaluation of delivering choice

Robust evidence about the effectiveness of interventions is ideally obtained from randomised controlled trials to reduce confounders, but identifying and recruiting a suitable comparison group is difficult with the dying. Delivering Choice projects have been the subject of several previous evaluations [12-15]. Using difference in difference analysis, a quantitative approach comparing geographical areas, an evaluation of the Lincolnshire Delivering Choice project found that Delivering Choice had a positive impact on decreasing NHS costs [15] while other evaluations of Delivering Choice projects were interview studies conducted with professionals about barriers and levers to implementation [12-14].

For this Delivering Choice evaluation, the funders (Marie Curie Cancer Care) wanted to know what worked and why with a control group for comparison. One aspect of this evaluation used routine data (e.g. hospital and service referral data) to investigate differences between Delivering Choice users and non-users in terms of deaths in hospital and hospital utilisation over a six month period from 1 September 2011 – 29 February 2012. By linking hospital admissions, place of death, ambulance and emergency (A&E) attendances and Delivering Choice service usage and controlling for confounders such as age, diagnosis and deprivation, we found that fewer Delivering Choice users died in hospital and had fewer emergency hospital admissions and A&E attendances at 30 or 7 days before death than those who did not access Delivering Choice services. Specifically, 67-80% fewer Delivering Choice users died in hospital and Delivering Choice users had 39-51% fewer hospital admissions 30 days before death which improved to 68-80% fewer hospital admissions in the last seven days. There were 34-59% fewer A&E attendances 30 days before death amongst Delivering Choice users and 68-78% fewer in the last seven days. Moreover, qualitative interviews with family carers suggested that service satisfaction was high and family carers and professionals reported excellent care coordination. Table 2 provides details of the quantitative findings; these results have been published elsewhere [16] (Table 2).

**Table 2:** Details of findings from quantitative analysis of routine data

	**North Somerset**		**Somerset**	
	**OR**	**95% CI**	**OR**	**95% CI**
Hospital deaths	0.33	0.20, 0.50	0.20	0.17, 0.27
Emergency hospital admissions at 30 days	0.49	0.33, 0.75	0.61	0.48, 0.76
Emergency hospital admissions at 7 days	0.22	0.12, 0.44	0.32	0.23,, 0.45
A&E attendances at 30 days	0.41	0.28, 0.62	0.66	0.51, 0.85
A&E attendances at 7 days	0.22	0.11, 0.42	0.32	0.22, 0.67

For the second aspect of this evaluation, realist evaluation was employed to find out ‘what worked for whom and why’ to explore why Delivering Choice users more often died at home and appeared to use fewer hospital services. We wanted to identify the helpful (and unhelpful) factors that contributed to these quantifiable outcomes and high family carer satisfaction. The results from this second aspect are presented in this paper.

## Methods

### Study design

With realist evaluation, the key question is ‘what works for whom and in what circumstances?’ Working hypotheses are generated made up of Context-Mechanism-Outcome (CMO) configurations. Outcomes are defined as intended or unintended consequences; ‘mechanism’ is defined as what brings about a change in behaviour and context is the circumstances that may affect whether and to what extent the mechanism is triggered [17]. Context is about having the right conditions to activate the mechanism. Once developed, these configurations are tested to develop ‘middle range theories’ to explain how and why particular interventions work. With further testing, these hypotheses are then generalisable to other contexts.

### Data collection

An important tenet of realist evaluation is making explicit the assumptions of the programme developers. Over two dozen local stakeholders attended three ‘hypothesis generation’ workshops before fieldwork started, where many CMO configurations were proposed. These were then tested through data collection of observations, interviews and documentation.

Fifteen formal observations of all Delivering Choice services occurred at two time points: 1) baseline (August 2011) and 2) mid-study (Nov-Dec 2011). These consisted of a researcher sitting in on training sessions facilitated by the End of Life Care facilitators with care home staff and shadowing Delivering Choice staff on shifts at the Out of Hours advice line, Discharge in Reach service and coordination centres. Researchers also accompanied the personal care team on home visits on two occasions. Notes were taken while conducting formal observations and these were typed up and fed into the analysis.

To gather personal accounts of the services, we interviewed:

• 43 family carers and service users

• 11 staff delivering or managing Delivering Choice services

• 94 staff eligible to use the services including those who did and did not refer

To identify family carers and patients, we asked service providers to select service users from each quarter from when the service was launched. Patients and family carers were recruited and consented to the study by the Delivering Choice service providers and re-consented by the research team. We received written consent from all face to face participants and recorded verbal consent from all telephone respondents. To identify professionals using the services, we used referral databases. To identify those not using the services, we obtained lists of staff from different organisations (e.g. community nursing teams, GP practices, care homes). We also used snowball sampling, whereby previous interviewees identified other colleagues that might have useful information. For GP practices and care homes, we called organisations at random. With community nursing teams, two community nurses from each team were selected (one at senior and one at junior level).

Specific topic guides were developed for patients/family carers and professional groups and reviewed throughout the data collection. For example, family carers were asked how they heard about the service, what prompted its use, the quality of the service, whether the patient died in his or her preferred place of care and the contribution that the service made to facilitating death in place of choice. Professionals were asked similar questions and to recount examples of previous service use or situations in which service usage was considered and discarded. These fuller narratives were especially fruitful.

Details on the professions and type of interviews are presented in Table 3. All but three patient and family carer interviews were face to face, while interviews with professionals were by telephone and face to face. Some informal interviews took place while researchers carried out observations. All face to face interviews were audio recorded, anonymised and transcribed while notes were taken during telephone interviews and typed up after.

**Table 3:** Interview participants and type of interview

**Profession**	**Telephone**	**Face to face**	**Informal**	**Total**
Community nurses	16	6	0	22
Hospital nurses	3	2	13	18
Community palliative care nurses	4	7	4	15
Hospice clinical and administrative	14	0	0	14
Delivering Choice providers	2	9	0	11
Care home	5	2	0	7
General Practice surgery	5	0	0	5
General Practitioners	1	3	0	4
Ambulance	2	0	2	4
Community hospital	1	0	2	3
Hospital consultants	0	0	2	2
Total	53	29	23	105

To corroborate accounts from interviews and interpretations from observations and track the development of the different services, we collected referral forms, service specifications, monthly returns on referrals from services, reports from the local Marie Curie team and meeting minutes. We also extracted data from a hospice database which recorded service referrals to the advice line.

### Data analysis

To analyse this volume of data, we used framework analysis which is a deductive approach suitable for studies with large datasets and limited analysis time [18]. For each intervention, team members (GL, JP, BS, LD) familiarised themselves with the data s/he had collected such as observation notes, interview transcripts and written documents such as service specifications, meeting minutes and reports. We designed a proforma with themes for this study, using realist evaluation concepts i.e. ‘what helps to make it work?’ and ‘what prompts someone to use it?’ (Table 4).

**Table 4:** Proforma questions

1	How is it supposed to work?
2	How does it actually work?
3	What helps to make it work?
4	What makes it more difficult?
5	What would make it work better?
6	What prompts someone to use it?
7	Does it duplicate something else that’s already there?
8	What are the positive impacts?
9	What’s its impact on the evaluation outcomes of:
	a. Co-ordinated care
	b. Patient dying in place of choice?
	c. Hospital usage (ie admissions, A&E)
10	What are the unintended consequences?
11	What do patients/family carers think about it?
12	What else do we still want to know?
13	Any other comments?

Team members then ‘indexed’ the data [19] by allocating relevant sections of data. They completed a proforma report for each intervention. Team members submitted their proforma reports which were complied into a ‘master’ document using the same themes for each intervention. For this master report, data from the reports of each researcher were compared and contrasted, returning to original source data when needed, to identify commonalities and discrepancies.

In addition, we analysed the hospice database for the telephone advice line to elaborate findings and understand more about the service. We randomly sampled 10% of the population over the six month study period who had used the advice line. We conducted content analysis for each patient on source of referral, condition of patient, number of calls, motivation for call and next steps. All non-cancer patients were also identified, as the funder had a particular interest in non-cancer patient service usage, and we repeated the same analysis. We then combined the analyses from the 10% sample and non-cancer patients (total n = 58) and compared these against the narratives of family carers and patients who took part in interviews to check for interview selection bias.

In interpreting findings, the whole team, including SP who offered clinical insight as a GP, met every week over a three month period. In particular, disconfirmatory data that were different from the rest of the findings were identified and discussed and when necessary further data were collected. After these discussions, the ‘master’ report was re-drafted and discussed again in a further team meeting until consensus was reached. Preliminary findings were shared in with Delivering Choice service providers before the final report was drafted.

### Ethics

As a service evaluation, this study did not require NHS ethical approval. However, ethical approval was received from the Faculty of Medicine at the University of Bristol.

## Results

The results from each service will be presented and a summary statement encapsulating the ‘CMO configuration’, or working hypothesis, for each service concludes each section.

### North Somerset end of life care facilitators

The End of Life Care facilitators raised awareness of end of life care in North Somerset for health professionals. These two former mid-grade community nurses, who were experienced in end of life care, were appointed full time for 18 months, which was then extended. Their aim was to raise the profile of end of life care across the county and train staff across organisational boundaries in hospices, care homes, general practices and community wards. Given the number of different organisations and the size of the county, this was ambitious.

In response to staff requests for help or their own observations that a professional group was in need, the End of Life Care facilitators provided educational sessions and advice. They directed staff to other useful services. They also oversaw strategic changes in end of life care service delivery (e.g. the introduction of ‘just in case’ boxes). In practice, the nurses undertook any task that needed doing. Although their flexibility, responsiveness and willingness to offer support was valued, they lost the ability to prioritise because the scope of their responsibilities became too broad. Moreover, as community nurses, they found it difficult to access general practice (GP) surgeries, as GPs preferred to work with fellow GPs. However, the interactive training sessions, whereby participants considered their own death, appeared powerful in changing attitudes and behaviours amongst community nursing and care home staff. These staff gained confidence in working with the dying.

When I first started here 12 years ago the carers that used to work here, they were always frightened of going into a room if somebody was dying in case they were dead when they walked in. But now, they are a lot more confident in dealing with that and dealing with relatives that are crying or upset. (Care home matron)

A GP who had been practising for nearly two decades recognised that directly attributing changes in deaths in the community to the End of Life Care facilitators was difficult, but she had noticed that staff were more confident and better prepared to help facilitate deaths in the community.

It used to be kind of roughly 50/50 in acute hospitals and in the community and now it’s probably more like about 80-85% of our patients dying either at home or in nursing homes and so for me that’s really positive. Whether you can kind of directly make the link between what [the End of Life Care facilitators] have been doing…or whether it’s just patients just choosing that more I don’t know…but I’ve been here for about eighteen years now and I’ve definitely seen that huge shift…in the last few years towards staff feeling more confident and being better prepared and anticipating patients dying at home and the quality of dying at home or their care home. (GP GI)

With their willingness to help with any difficulty and through offering advice, direction and formal educational sessions, these highly skilled nurses with dedicated time (context) increased the confidence of professionals working with the dying (mechanism), which may have helped facilitate home deaths and fewer hospital admissions (outcome).

### North Somerset end of life care coordination centre and specialist personal care team

The usefulness of the End of Life Care facilitators was enhanced by collaboration with the North Somerset End of Life Care Coordination Centre, which the facilitators promoted to professionals from hospices, community wards and care homes. The Coordination Centre organised packages of care with equipment, night nurses/night sitters and personal care staff for patients dying at home. These patients were referred by community or hospice nurses. During the six month evaluation period, 15% of patients (153/1022) had contact with the North Somerset Care Coordination Centre, of whom about a third died from non-cancer related causes [10].

The North Somerset Coordination Centre was located on local authority premises. It was a ‘one stop shop’ with assistance in both financial assessment and care provision. The Coordination Centre was staffed with two nurses, three administrators and a nurse co-ordinator, who approved applications for ‘fast track’ funding. Fast track funding confers extra money from Continuing Healthcare to buy in palliative support services for those with healthcare needs expected to die within a specific timeframe, usually 6–8 weeks. The Coordination Centre also had its own in-house care team that offered personal care and psychosocial support to family carers and patients. This maximised flexibility to respond to changing patient and family needs. Family carers who were allocated the in-house personal care team appeared to gain reassurance in supporting the dying relative.

This patient is very close to death and has told the Generic Support Worker that she is ‘ready to die’, which the daughter knows but is anxious about. The Generic Support Workers engaged in a broad discussion of points raised by the daughter, covering social, work and family matters. This discussion appeared to help the daughter relax. (Observation notes 21.2.12)

However not all patients received this high level of service. The in-house personal care team could only provide services for a few patients and families (28 over a 101 day period) [10]. Other families were allocated private agency staff, with whom the Coordination Centre staff had limited communication, once the care package was put in place.

Some patients and carers stated that without the help of the Coordination Centre, the dying individual would have been admitted to hospital.

Family carer: [My wife] would have to be in a hospital or hospice or something [if care package not put in place]…Patient: Yeah, I wouldn’t be able to stay at home, no way. (Family carer MR and Patient RR)

Others mentioned how “comforting” they found the support offered.

They admitted her [mother] and they said she had a chest infection…Well [after] about ten days they [the hospital] said she was fit enough to go home…She wasn’t happy to come home, she didn’t feel confident to go home and I wasn’t overly happy so she said could I find somewhere for her to go for a week or two respite? So I got her into [residential care home]…she was very, very poorly… She [Coordination Centre nurse] got like a hospital bed arranged to go in and obviously the Rapid Response she got to go in because they were the only ones that could administer the morphine…She said ‘I will stay on until I’ve got everything set up’…She came back quite later in the evening and said yes everything had been set up and yes it had been funded, so there is no problems with that…I was really pleased, I’ve never had any experience before but how efficient she was and got everything all set up to me so smoothly, it was quite comforting. (Family carer NR)

The North Somerset Coordination Centre efficiently and quickly allocated appropriate, flexible care packages. This was possible with their model of including a ‘fast track’ nurse coordinator to assess financial needs and an in-house dedicated personal care team (context). Those who used this Coordination Centre were “comforted” and reassured (mechanism). This may have contributed to their family carers’ capacity to support a community based death and avoid hospital utilisation and high levels of family carer satisfaction (outcome).

### Somerset end of life care coordination centre

Although the Somerset End of Life Care Coordination Centre had the same remit of organising care packages as the North Somerset Care Coordination Centre, only ‘fast track’ patients with approved funding were eligible. Eleven percent (294/2572) of Somerset palliative patients in our study used this service. Of those, a remarkable 70% died at home and 25% died from causes other than cancer [10]. The service model was different from the one in North Somerset as the Somerset Care Coordination Centre was led by a nurse manager and staffed by four administrators, without its own personal care staff team, additional nurses or ‘fast track’ nurse co-ordinator. To make this model successful, the Somerset Coordination Centre relied on good external relationships, particularly with referring community and palliative care nurses, care agencies and the fast track team. These relationships were further enhanced as this Coordination Centre systematically telephoned all relevant professionals (and families if appropriate) whenever a change in a care package occurred. The enthusiasm of this team and the high level of interpersonal skills of the nurse manager were particularly noteworthy. Advantages of this model were that it cost less than the North Somerset Coordination Centre and they employed night staff on contract. This meant that the chances of allocating seven night sits a week increased, which was vital to those dying at home without the support of family.

Community palliative care nurses were enthusiastic about Somerset Coordination Centre as it reduced the time spent organising care packages and freed them from the “worry” when those care packages failed.

You can always check with the Coordination Centre. And if patients phone in and say their night sit didn’t turn up or carers didn’t turn up, they [Coordination Centre] will get on to the agency and if one agency can’t help then they know other agencies they can go to. So yeah, that just takes a whole lot of worry out. It’s a box we can tick with confidence. (Community palliative care nurse RT)

Moreover, some community staff attributed the contribution of the Somerset Coordination Centre with more people dying in the community.

I think even sort of ten years ago there weren’t that many people that…you could actually facilitate them to actually stay at home for end of life care at all. And the equipment and things that we can get available now and get in there to actually help them…I don’t know what the statistics are but I’m sure that they must be very different ten years ago to what they are now for actually people staying at home and being cared for at home now, it must be a huge difference. (Community palliative care nurse LC)

The Somerset Coordination Centre was efficient in supplying and monitoring comprehensive care packages, including night sits. It was staffed by a capable team that developed extensive, good quality personal relationships with relevant professionals (context). Those who used the Coordination Centre were freed from “worry” and able to access the provision needed (mechanism). This supported deaths in the community (outcome).

### Somerset out of hours advice and response telephone line

Managed by the local hospice, the Out of Hours advice and response line was manned by an experienced palliative care nurse on weekday evenings until 1 am, on weekends and bank holidays. This nurse had no other duties other than answering the telephone to respond to calls from patients, family carers and professionals. After 1 am, calls went to the night nurse on duty at the hospice ward. Over the six month period, the referral database included 1029 calls for 391 patients, of whom 18% died from conditions other than cancer. The quantitative analysis suggested that the advice line was particularly useful in the last week of life. Offering advice, support and triaging, use of this service was often triggered by a patient crisis, professional or family carer uncertainty and/or requests for urgent care visits. This service capitalised on the success of the daytime telephone line run by the hospice by using the same telephone number. Almost all of the patients were already known to the hospice and so good quality electronic data on the patients were available when the call was received.

Of value to family carers was the call back to families from the advice line nurse a few hours after a crisis.

On one occasion…[husband] was in such awful pain… So, in desperation, I rang my GP…but they were just going off duty and said…ring the [on call doctor]… and I was so unhappy about it, I thought a strange doctor will come here, they won’t know his history and…I rang the hospice Out of Hours and I spoke to a lady called [X], ..she went and got his records…she said ‘Just hang on, I know about this case’ and then she told me exactly what to give him… and then he became calmer…and he was out of this awful agony and I felt so relieved…and then what was most amazing and lovely, about an hour later, she range me back and she said ‘How are things?’.. I’ve never been so grateful to anyone in my life. (Family carer HJ)

Patients could also ring the advice line to receive reassurance that their experiences were “normal”.

[My husband (patient)] would sometimes phone [Advice Line] just to say “This is happening…what should be happening? … This is what I’m feeling” and so it was reassurance for him as well. (Family carer JM)

Some family carers and patients attributed their use of the out of hours advice line to a reduction in use of paramedics, as there was now an alternative during a crisis.

I didn't ever have to phone for ambulances or anything, all that was done and it wasn't done through the GP or the district nurse… you could just phone one number [OOH Advice Line]…and then they would get you sorted. (Family carer MI)

The advice line offered twenty four hour access to experienced palliative care nurses. These nurses offered advice and support and had access to electronic information about the patient (context). Those who contacted the Advice Line, often in a crisis, were reassured (mechanism). This contributed to high family carer satisfaction and less use of paramedics and Accident and Emergency departments (outcome).

### Discharge in reach nursing service

The Discharge in Reach nursing service operated in two hospitals. Over the six month study period, 144/2572 (5.6%) of the total eligible population in Somerset received this service. Forty percent died from non-cancer conditions, the highest of any service. The ‘in reach’ component meant that the nurses identified their own caseload, rather than accepting referrals from elsewhere. This might account for the larger proportion of non-cancer patients.

Two nurses (one in each hospital) were placed in Medical Admissions Units, Surgical Admission Units and emergency departments to identify and discharge patients for whom there would be no further benefit from medical treatment. These patients did not want to die in hospital. These skilled nurses supported patients and family carers in making decisions about preferred place of care and requested care packages to facilitate deaths in the community. These care packages were set up by Somerset Coordination Centre and the Coordination Centre was key to the success of the Discharge in Reach nurses in facilitating quick discharges. Importantly, the Discharge in Reach nurses also challenged consultants about unnecessary treatments and probed family carers who had unrealistic notions about supporting the dying at home. Several participants noted how highly skilled these nurses were in conducting difficult, sensitive discussions with senior clinical colleagues, family carers and patients. The nurses themselves believed that being dedicated to this role, without other duties and with sufficient time for each patient/family, was crucial.

The Discharge in Reach nurses had an educational remit to increase the skills and knowledge of hospital staff in end of life care, particularly around the availability of community services, to improve staff confidence around patient discharge.

[The Discharge in Reach nurse] is able to explain in an invaluable way the details of how patients can be supported in moving back into the community from hospital and receiving appropriate care there…The ward sister told me that [the Discharge in Reach nurse] helps staff gain confidence, adding “she has given me that confidence and that knowledge” regarding patient discharge. (Observation notes, 7.12.11)

This increased staff confidence in their own skills. The knowledge that appropriate care was available in the community may have contributed to hospital staff being willing to discharge the dying from hospital.

Certainly in the past if you lived at home on your own and you appeared to be in the last few weeks of life the chances of you getting discharged must have been zero really but I’ve certainly seen people that [the Discharge in Reach nurse] has facilitated that to happen for. (Community palliative care nurse RT)

These nurses had dedicated and sufficient time to identify patients wanting to die at home who were in hospital settings. The nurses carried out sensitive, difficult conversations with clinicians and family carers. They could then allocate appropriate care packages (with the help of the Coordination Centre) (context). The highly skilled Discharge in Reach nurses facilitated quick discharges which contributed to more people dying in the community (outcome). These nurses also increased the confidence of hospital staff (mechanism) in allowing discharges, even for those patients who lived alone.

## Discussion

### Limitations and strengths

In terms of strengths, realist evaluation methodology was helpful when faced with multiple avenues of enquiry and large volumes of data, as we revisited ‘what works for whom and in what circumstances?’ We collected accounts from patients, family carers and professionals from various backgrounds (hospital, community, hospice), observed services at different time points and gathered copious documentation. Ideally we would have liked to audio record more interviews, but as with many service evaluations the short time frame made this difficult. However through iterative cross comparisons of multiple data sources, the robustness of our findings were strengthened. Furthermore, as a ‘real world’ service evaluation this study has valuable, applicable information that commissioners and policy makers can utilise to inform their decision-making, in line with recommendations from the End of Life Care Strategy 2008 [1]. In fact, findings from this study have already directly fed into the development of a new End of Life Coordination Centre in Bristol.

Generalisability (or lack thereof) is a particularly contentious issue within qualitative research [20-22]. Qualitative research is not generalisable in the same way as quantitative research, because the reader, not the researcher, undertakes the transfer processes. However, qualitative research offers the opportunity for what Stake calls “naturalistic generalization”, whereby readers “find descriptions that resonate with their own experiences…[and then] transfer knowledge from a study sample to another population” [23]. Realist evaluation offers a halfway house between quantitative generalisability and qualitative transferability through the generation of concrete, explanatory ‘middle range’ theories suitable for testing in future studies. Each ‘CMO configuration’ stated in the concluding paragraphs for each service in the results section offers opportunities for further testing of findings.

### Summary of results

In summarising findings across the Delivering Choice programme, we found that highly skilled, experienced, customer-focused palliative professionals with dedicated and sufficient time were important. These staff were willing to have difficult conversations about death and the practicalities of caring for the dying with family carers, patients and/or clinical colleagues (context). They had access to resources in the community to support homecare and delivered excellent services (context). This engendered confidence and reassurance to staff, family carers and patients (mechanism). The reassurance for patients and family carers stemmed from validation that their experience was ‘normal’ and that experienced care givers, often who knew the patents well, were available in case of emergency (mechanism). The confidence for staff came from their improved skills in working with those at the end of life and knowledge that their patients would be well looked after (mechanism). This may have contributed to high family carer satisfaction, low hospital utilisation and more deaths in the community amongst Delivering Choice service users (outcome).

Like many new services, what limited the success of Delivering Choice was sporadic, patchy use. Referring professionals often (erroneously) reported that services were only for cancer patients and/or those who received ‘fast track’ funding. Possibly, due to confusion about these eligibility criteria, results from routine data found that in Somerset less than a quarter of all potential patients accessed Delivering Choice services (616/2572). In North Somerset that fell to just over a fifth (213/1022). The use of services amongst those with conditions other than cancer ranged from 18-40%. Moreover, ways to identify the dying early in their trajectory were limited and so the median time to death for service usage was 6–20 days [10]. This suggests that although of excellent quality and successful in steering its users away from hospitals, Delivering Choice was mainly ‘working’ for those with cancer who had obtained fast track funding and were close to death. To really harness the potential of Delivering Choice services, strategies need to be developed and implemented to reach non-cancer patients and those at earlier stages of the dying trajectory. This appears particularly possible with the location of Coordination Centres within social services on local authority premises, as early warning systems could be set up with those requesting social care packages. Services where the providers identified their own caseload (i.e. Discharge in Reach nurses) also had the highest success rate in targeting non-cancer patients.

Our results suggest that the success of Delivering Choice appears to partially rely on charismatic, talented staff. The enthusiasm and attitudes of these professionals may be eroded by threats to the stability of funding, re-organisations, policy changes, burn-out, resignations, fewer staff etc. In fact, we witnessed this at a ‘Celebration Evening’ with the service providers, funders and commissioners in December 2012. The effects of the Health and Social Care Act 2012, which have resulted in a major re-organisation of the National Health Service, were already in evidence. In six short months since completing fieldwork, there appeared little celebration and much dismay at the changes to the services and staff morale. However, the success of these services was not entirely due to charismatic staff, as the original team from one service experienced poor leadership and low morale, but the quantitative data still suggested that the service was successful, as services users visited or died in hospital less frequently. Other factors such as delivering an efficient service and having access to resources to support homecare appeared sufficient (in this case) to generate reassurance amongst family carers and confidence amongst referring staff. Given the complexity of end of life care service provision and the potential confounders in a non-randomised study, further research, especially into Coordination Centres which are being established across the country, would be useful, ideally using a mixed methods design testing the CMO configurations identified in this study.

### Wider implications

In considering the wider implications of these findings, ‘improving primary care management of end of life care’ was recently identified as one of the top ten priorities for Clinical Commissioning Groups, which are the organisations in England that allocate funding to services [24]. An approach was encouraged with:

• Facilitation of discharge from the acute system

• Centralised co-ordination of care provision in the community

• Guaranteeing 24/7 care

In evaluating Delivering Choice, we found evidence that the Discharge in Reach service facilitated rapid discharge from the acute sector with the support of the Somerset Coordination Centre. The Coordination Centres efficiently centralised coordination of care provision. Moreover, the Out of Hours advice line that was available 24 hours a day and went beyond giving advice to patients and family carers by resolving problems where possible. In many ways, Delivering Choice appears to meet these policy recommendations.

This is important, given the policy push to increase the numbers of those dying at home. The burden of homecare is on family members with little or no experience of the dying [25,26]. Family carers and dying patients need reassurance from trusted professionals with experience in palliative care, especially during a crisis, otherwise they turn to hospitals and emergency services. Some may argue that community nurses already fulfil this role. But community nurses are generalists, not specialists, with increasing demands on their time. Our study also suggests that community nurses also appreciated the confidence that came from working with the specialised support of experienced palliative care staff.

To be effective, palliative care professionals possessed explicit formal knowledge based on clinical experience, referral protocols and guidelines and, equally importantly, informal knowledge of health and social care systems to act on the behalf of families and patients. For example, the nurse manning the advice line knew that a particular community nurse would know where to check for a replacement for a faulty mattress at a local hospital. In another instance, a Discharge in Reach nurse set up a placement for a patient in a care home which could potentially provide wireless access for his laptop; she knew which care homes had this facility which was important to this particular patient. We would argue that few community nurses possess this sort of information across an entire health and social care economy.

With the rising demand for palliative care provision out of hours [27] and with the increasing fragmentation of service providers across the social and healthcare system [28], family carers and patients will have ever greater need of experienced, committed, proactive staff with this type of informal knowledge. They offer an inter-organisational overview across the voluntary, social care, acute and community healthcare sectors to help navigate patients and families through the system round the clock. Specialised end of life care services such as End of Life Care Coordination Centres, Out of Hours advice and response lines and Discharge in Reach nurses appear particularly successful in addressing this need. This will help to meet the policy objectives of greater home deaths.

## Conclusion

The findings from this particular evaluation of a major re-configuration of end of life care services suggest that in the early days of the initiative, the Delivering Choice Programme primarily ‘worked’ for those with cancer who received fast track funding. The success of high family carer satisfaction, low hospital utilisation and more deaths in the community appeared related to services staffed with highly skilled, experienced, enthusiastic palliative care professionals. These professionals had dedicated and sufficient time to engage in difficult conversations about death and the practicalities of caring for the dying with family carers, patients and/or clinical colleagues. They had access to resources in the community to support homecare and informal knowledge of how the system worked to deliver excellent services that engendered confidence and reassurance to staff, family carers and patients. As the demand for end of life care grows and services fragment across care sectors, there is potentially an important role for specialised, 24 hour integrated palliative services such as these which have an overview to help steer families and patients through the system.

## Competing interests

The authors declare they have no competing interests.

## Authors’ contributions

LW and SP were co-leads on this project. LW designed the realist evaluation, managed the research associates, carried out data analysis and drafted reports. GL and collected documentation and databases, carried out interviews and observations, conducted primary analysis and contributed to draft reports and papers. JP led on the interviews with patients and family carers, interviewed and observed staff, conducted primary analysis and contributed to draft reports and papers. LD carried out telephone interviews with staff, conducted primary data analysis, conducted analyses on the advice line database and contributed to draft reports and papers. BS analysed documentation and contributed to draft reports and papers. SP led on the quantitative aspect of the evaluation, contributed to weekly discussions of emerging findings from the realist evaluation and contributed to draft reports and papers. All authors read and approved the final manuscript.

## Pre-publication history

The pre-publication history for this paper can be accessed here:

http://www.biomedcentral.com/1472-684X/13/37/prepub
